# Supplementary material for: Improving Anti-Coking Properties of Ni/Al2O3 Catalysts via Synergistic Effect of Metallic Nickel and Nickel Phosphides in Dry Methane Reforming
Source: Materials (Basel). 2022 Apr 22;15(9):3044. doi: 10.3390/ma15093044 (PMC9101347; doi:10.3390/ma15093044)
Supplement: Supplementary file 1 [file materials-15-03044-s001.zip › materials-1684337-supplementary.pdf]

Supplementary information for

Improving Anti-coking Properties of Ni/Al_2O_3 Catalysts via the Synergistic Effect of Metallic Nickel and Nickel Phosphides in Dry Methane Reforming

Yu Shi, Shiwei Wang, Yiming Li, Fan Yang, Hongbo Yu, Yuting Chu, Tong Li* and Hongfeng Yin *

Ningbo Institute of Materials Technology and Engineering, Chinese Academy of Sciences, 1219 Zhongguan West Road, Ningbo, Zhejiang 315201, P. R. China

*Correspondence: litong@nimte.ac.cn, yinhf@nimte.ac.cn;

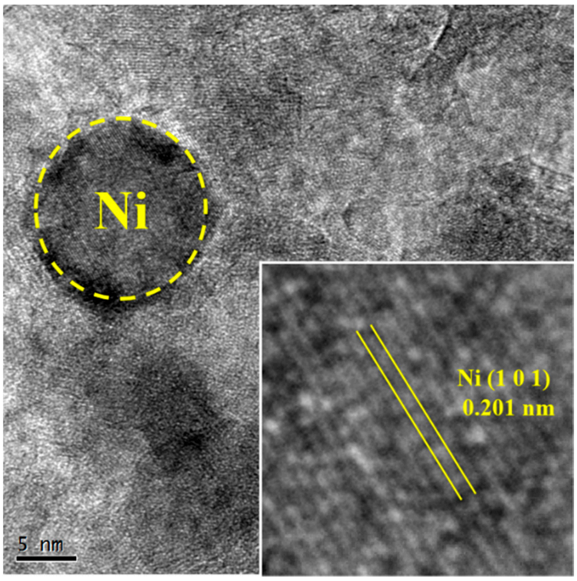

Figure S1. HRTEM image of the reduced Ni/Al_2O_3 catalysts (the inset is the magnified image of Ni).

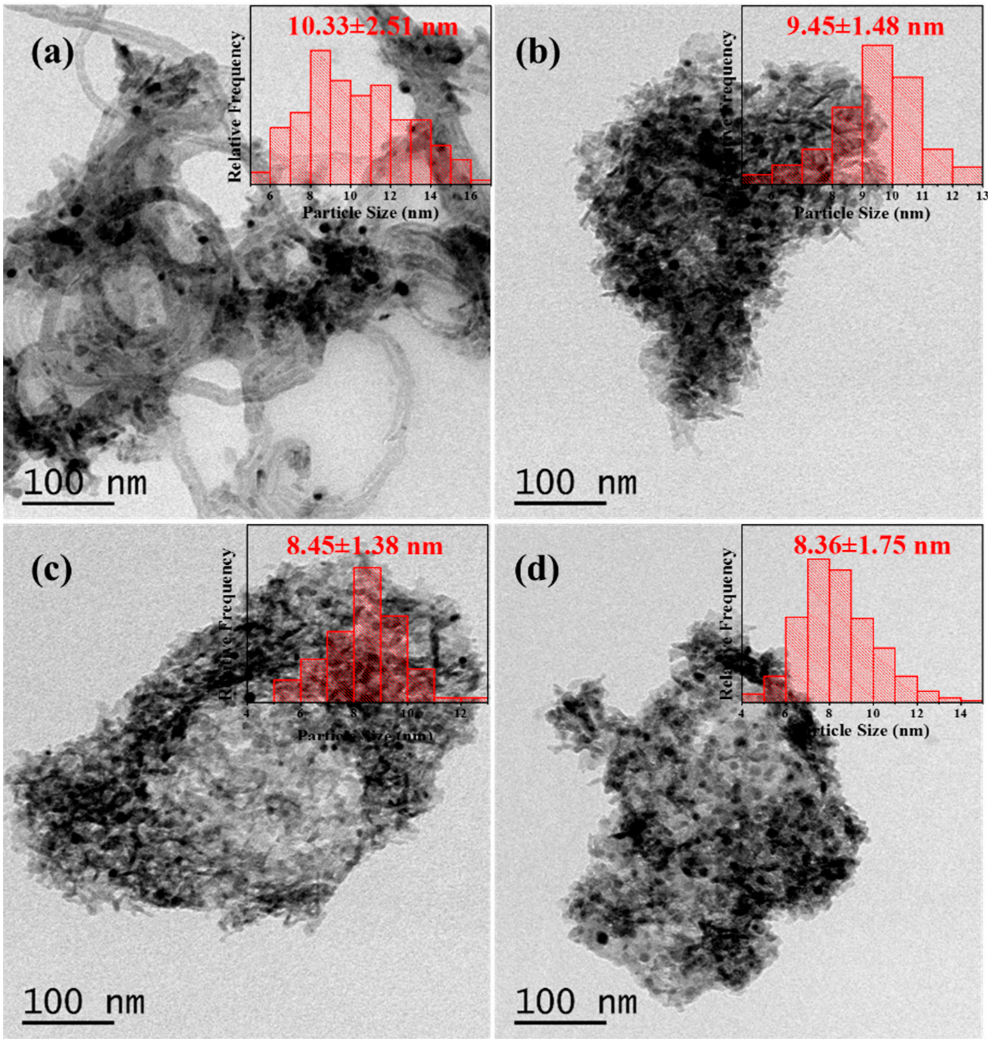

Figure S2. TEM images and corresponding size distributions of spent (a) Ni/Al_2O_3; (b) NiP-4/Al_2O_3; (c) NiP-3/Al_2O_3; (d) NiP-2/Al_2O_3.

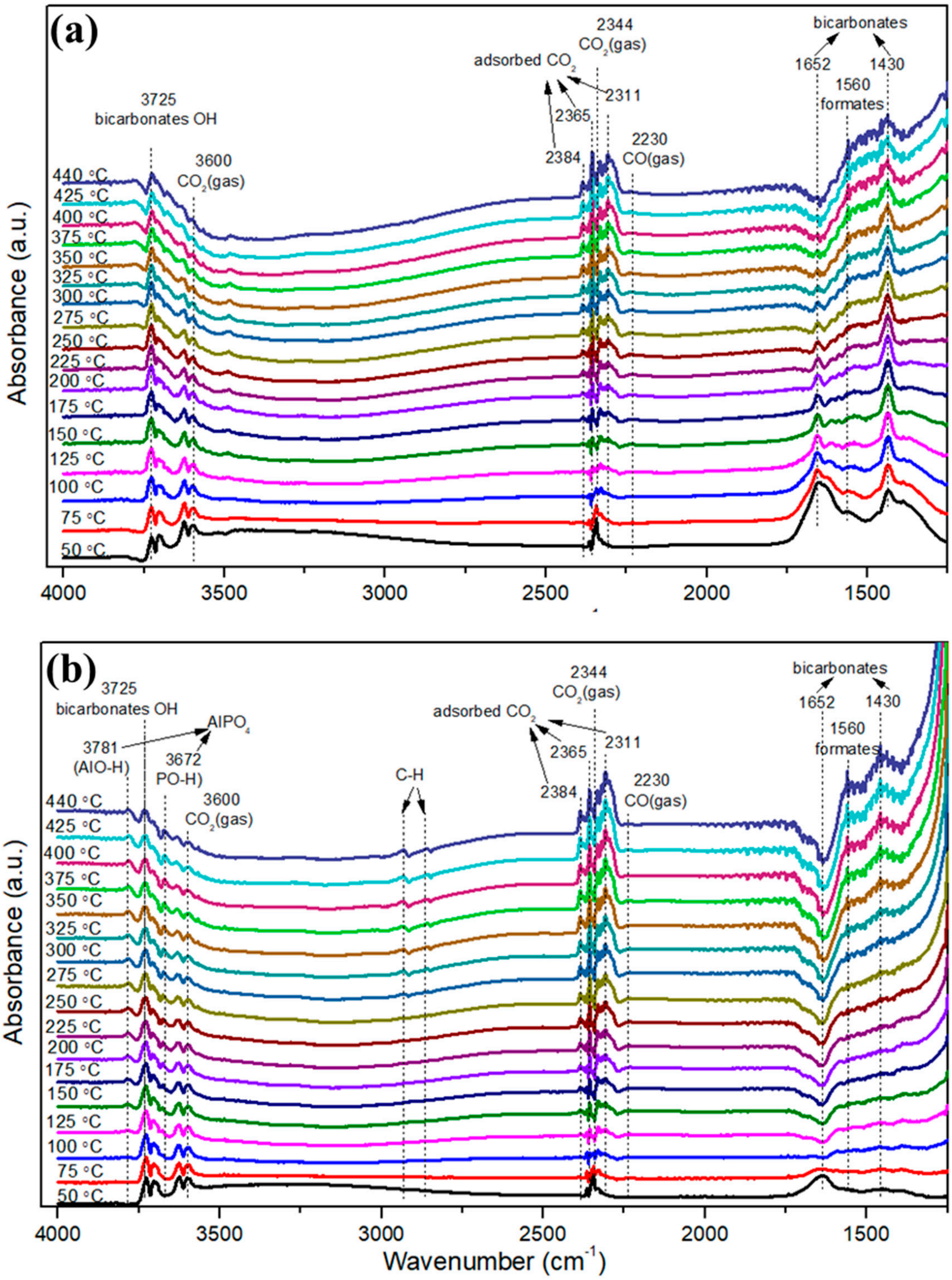

Figure S3. DRIFTS spectra of CO_2 temperature programmed adsorption at different temperatures over (a) Ni/Al_2O_3 and (b) $NiP\text{-}2/Al_2O_3$.
